# Single Exposure to the Cathinones MDPV and α-PVP Alters Molecular Markers of Neuroplasticity in the Adult Mouse Brain

**DOI:** 10.3390/ijms22147397

**Published:** 2021-07-09

**Authors:** Lucia Caffino, Francesca Mottarlini, Sabrine Bilel, Giorgia Targa, Micaela Tirri, Coralie Maggi, Matteo Marti, Fabio Fumagalli

**Affiliations:** 1 Department of Pharmacological and Biomolecular Sciences, Università degli Studi di Milano, Via Balzaretti 9, 20133 Milano, Italy; lucia.caffino@unimi.it (L.C.); francesca.mottarlini@unimi.it (F.M.); giorgia.targa@unimi.it (G.T.); coralie.maggi@unimi.it (C.M.); 2 Section of Legal Medicine and LTTA Center, Department of Translational Medicine, University of Ferrara, 44121 Ferrara, Italy; sabrine.bilel@unife.it (S.B.); micaela.tirri@unife.it (M.T.); mto@unife.it (M.M.); 3 Collaborative Center for the Italian National Early Warning System, Department of Anti-Drug Policies, Presidency of the Council of Ministers, 44121 Ferrara, Italy

**Keywords:** GABA, glutamate, NPAS4, BDNF, MDPV, α-PVP, cathinones, hippocampus, frontal cortex, mouse

## Abstract

Synthetic cathinones have gained popularity among young drug users and are widely used in the clandestine market. While the cathinone-induced behavioral profile has been extensively investigated, information on their neuroplastic effects is still rather fragmentary. Accordingly, we have exposed male mice to a single injection of MDPV and α-PVP and sacrificed the animals at different time points (i.e., 30 min, 2 h, and 24 h) to have a rapid readout of the effect of these psychostimulants on neuroplasticity in the frontal lobe and hippocampus, two reward-related brain regions. We found that a single, low dose of MDPV or α-PVP is sufficient to alter the expression of neuroplastic markers in the adult mouse brain. In particular, we found increased expression of the transcription factor *Npas4*, increased ratio between the vesicular GABA transporter and the vesicular glutamate transporter together with changes in the expression of the neurotrophin *Bdnf*, confirming the widespread impact of these cathinones on brain plasticity. To sum up, exposure to low dose of cathinones can impair cortical and hippocampal homeostasis, suggesting that abuse of these cathinones at much higher doses, as it occurs in humans, could have an even more profound impact on neuroplasticity.

## 1. Introduction

Plasticity is the ability of an organism to appropriately respond to an external stimulus through the activation of transcriptional mechanisms set in motion by neuronal activity that, in turn, activate intracellular signaling pathways thus fostering the induction of immediate early genes (IEGs). IEGs, then, promote the activation of downstream targets leading to functional and structural changes in the brain [1,2]. A recently discovered, neuronal-specific transcription factor, known as NPAS4, has emerged as a key mediator of brain plasticity. NPAS4 is regulated in an activity-dependent fashion and it has proven essential for the development of GABAergic synapses onto excitatory neurons [3]. One of the intrinsic features of NPAS4 appears to be its homeostatic ability to tone down neuronal firing in response to excitatory transmission, by potentiating inhibitory transmission [3]. This is critical since a proper balance between excitation and inhibition is relevant for the function of neuronal networks and, therefore, the regulation of NPAS4 appears to contribute to neuroplasticity as, for instance, knockout mice lacking this gene exhibit features reminiscent of social anxiety [4] and cognitive deficit [5]. Interestingly, among its different characteristics, it also has to be taken into account that NPAS4 itself is able to control genes that, in turn, regulate the development of inhibitory synapses, some of them positively and some other negatively, thus widening the role of *Npas4* as a transcriptional repressor/activator [3]. Accordingly, the regulation of NPAS4 expression may have a large downstream impact on neuroplasticity.

Along this line of reasoning, among the genes that are regulated by NPAS4, a critical role is indeed played by Brain Derived Neurotrophic Factor (BDNF), which is known to contribute to the development of GABAergic synapses [6,7,8]. BDNF is a neurotrophin that plays a pleiotropic role in the central nervous system (CNS). Classically, its primary role was confined during CNS development for the regulation of cell growth, cell survival and cell differentiation [9], whereas, more recently, BDNF has been demonstrated as a master regulator of neuroplasticity, as shown by its critical role in activity-dependent structural remodeling [10] as well as in the modulation of cognition [11]. Of note, both NPAS4 [12,13] and BDNF [14,15,16,17,18] have been involved in the actions of drugs of abuse, primarily psychostimulants. These data suggest that, due to their respective features, both proteins may be important in the action of psychostimulants. Accordingly, a role can be hypothesized for both of them in the action of synthetic cathinones, a recently emerging class of psychostimulant designer drugs used for their rewarding properties that are similar to cocaine, methylenedioxymethamphetamine or other psychoactive drugs, such as amphetamine and amphetamine-like molecules [19,20,21]. While these compounds were initially commercialized through Internet or smart shops as’ bath salts’ or ‘plant foods’, nowadays they are sold under their own chemical name in the clandestine market [22,23]. In humans, MDPV and α-PVP are primarily used orally, by snorting (insufflation) or smoking, but also through intravenous injection (« slam ») or buccal, sublingual, and rectal applications [24,25]. Among the several synthetic cathinones existing on the clandestine market, we focused our attention on the methylenedioxy derivative of pyrovalerone MDPV (3,4-methylenedioxypyrovalerone) and its closely related derivative α-PVP ((1-Phenyl-2-(pyrrolidin-1-yl)pentan-1-one), also known as “flakka” or “gravel”) [26,27,28,29,30]. While synthetic cathinones exhibit features typical of amphetamines-like compounds, MDPV and α-PVP more closely resemble cocaine-like compounds [31] as they primarily block the dopamine transporter (DAT) and the norepinephrine transporter (NET), whereas, at variance from cocaine [32,33], these two compounds do not seem to block the serotonin transporter (SERT) [34,35,36,37,38]. Further, MDPV and α-PVP show reinforcing properties higher than cocaine and their abuse liability is positively correlated with their ability to block the DAT [39]. Interestingly, the removal of DAT has been associated with changes in the expression of the neurotrophin BDNF in cortical brain regions [40,41]. Above all, in rodents, their effect is manifested by increased locomotor activity, presence of stereotypies, appearance of psychomotor effects [28,42,43,44,45] as well as aggressive behaviors, more severe than those caused by cocaine and other psychostimulants such as amphetamine, methamphetamine and methiopropamine administration [46,47,48]. Besides these behavioral manifestations, they display reinforcing and rewarding properties in rodents [28,42,43,49,50,51]. 

Given these premises, the major aim of our study was to investigate the effect of a single exposure to MDPV or α-PVP on *Npas4* mRNA levels in the frontal lobe and hippocampus, two brain regions where this transcription factor is mainly expressed [3]. We have recently shown that both drugs modulate the expression of the immediate early genes *Arc/Arg3.1* and *c-Fos* in these brain regions following a single injection indeed suggesting that these synthetic cathinones cause changes in neuronal activity that, in turn, may affect neuroplasticity [52]. In addition, we have also shown that a single injection of the psychostimulant cocaine is sufficient to provide functional changes in the hippocampus [53] and in the prefrontal cortex [54]. 

We will couple the information deriving from the analysis of *Npas4* mRNA levels with the evaluation of a marker of neuroplasticity, the neurotrophin BDNF, known to be regulated by NPAS4 as above mentioned. Further, given the necessity of NPAS4 for the development of GABAergic synapses onto excitatory neurons [3], we will investigate whether the acute action of cathinones is sufficient to alter the coordinated balance between excitatory and inhibitory inputs. Accordingly, we will analyze the ratio between the vesicular transporters of both GABA (*vGAT*) and glutamate (*vGluT1*), whose roles are to package these neurotransmitters into vesicles in the cytoplasm, ready to be released following a stimulus [55] and that represent an index, albeit indirect, of the release of these neurotransmitters. Of note, evidence already exists for the role of *vGluT1* and *vGAT* in the action of psychostimulants [55,56,57].

## 2. Results

### 2.1. Molecular Analyses of the Effects of MDPV and α-PVP on Npas4 mRNA Levels 

#### 2.1.1. Hippocampus

**Figure 1:**
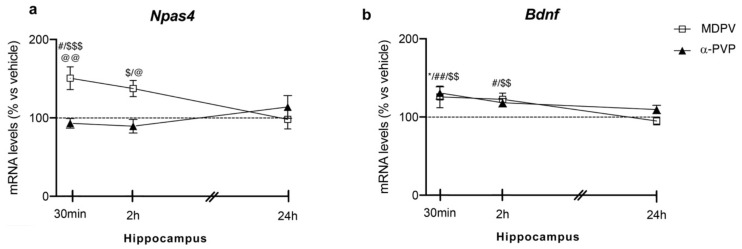
Time-dependent changes in *Npas4* (**a**) and *Bdnf* (**b**) mRNA levels in the hippocampus of mice following a single IP injection of MDPV (1 mg/kg) or α-PVP (1 mg/kg). Mice were sacrificed at three different time points, i.e., 30 min, 2 h, and 24 h after the drug administration. Results are expressed as percentages relative to vehicle-treated group and presented as mean ± standard error of the mean (SEM). * *p* < 0.05 α-PVP versus vehicle-treated mice; ^#^ *p* < 0.05, ^##^ *p* < 0.01 MDPV versus vehicle-treated mice; ^$^ *p* < 0.05, ^$$^ *p* < 0.01, ^$$$^ *p* < 0.001 MDPV versus MDPV-24 h; ^@^ *p* < 0.05, ^@@^ *p* < 0.01 MDPV versus α-PVP (two-way ANOVA followed by Tukey’s multiple comparisons test).

Two-way ANOVA on *Npas4* mRNA levels in the hippocampus showed a main effect of treatment (F_2,43_ = 8.774, *p* = 0.0006), time after injection (F_2,43_ = 6.828, *p* = 0.0027) and a significant time × treatment interaction (F_4,43_ = 3.935, *p* = 0.0083; Figure 1 panel A). In detail, post hoc analysis showed that the temporal induction of *Npas4* differs between the cathinones used and the time of sacrifice after injection. In fact, a single injection of MDPV up-regulated *Npas4* mRNA levels 30 min (+51%, *p* = 0.0285 vs. vehicle-30 min; +58%, *p* = 0.043 vs. α-PVP-30 min; +71%, *p* = 0.0002 vs. MDPV-24 h), 2 h (+46%, *p* = 0.0447 vs. α-PVP-2h; +51%, *p* = 0.0173 vs. MDPV-24 h), an effect that dissipated 24 h after the injection (+9%, *p* > 0.999 versus vehicle-24 h). Conversely, a single injection of α-PVP, instead, did not alter *Npas4* mRNA levels at any of the time points investigated (30 min: −7%, *p* > 0.999 versus vehicle-treated mice; 2 h: −10%, *p* = 0.997 versus vehicle-treated mice; 24 h: +11%, *p* = 0.997 versus vehicle-treated mice). 

#### 2.1.2. Frontal Lobe

**Figure 2:**
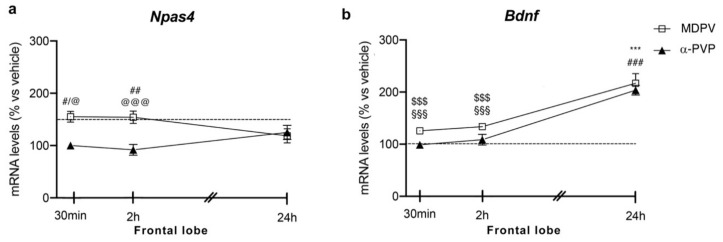
Time-dependent changes in *Npas4* (**a**) and *Bdnf* (**b**) mRNA levels in the frontal lobe of mice following a single IP injection of MDPV (1 mg/kg) or α-PVP (1 mg/kg). Mice were sacrificed at three different time points, i.e., 30 min, 2 h, and 24 h after the drug administration. Results are expressed as percentages relative to vehicle-treated group and presented as mean ± standard error of the mean (SEM). *** *p* < 0.001 α-PVP versus vehicle-treated mice; ^#^ *p* < 0.05, ^##^ *p* < 0.01, ^###^ *p* < 0.001 MDPV versus vehicle-treated mice; ^$$$^ *p* < 0.001 MDPV versus MDPV-24 h; ^@^ *p* < 0.05, ^@@@^ *p* < 0.001 MDPV versus α-PVP; ^§§§^ *p* < 0.001 α-PVP versus α-PVP-24 h (two-way ANOVA followed by Tukey’s multiple comparisons test).

Similar to the hippocampus, two-way ANOVA showed a main effect of treatment (F_2,43_ = 16.28, *p* < 0.0001) and a significant time × treatment interaction (F_4,43_ = 3.875, *p* = 0.0089) on *Npas4* mRNA levels (Figure 2 panel A). Again, post hoc analysis revealed a temporal induction of *Npas4*, which depends on the cathinone being analyzed and the time of sacrifice after injection. In fact, a single injection of MDPV upregulated *Npas4* mRNA levels 30 min (+55%, *p* = 0.0219 vs. vehicle-30 min; +55%, *p* = 0.0136 vs. α-PVP-30 min), 2 h (+61%, *p* = 0.0046 vs. vehicle-2h; +70%, *p* = 0.0007 vs. α-PVP-2h; +51%, *p* = 0.0322 vs. MDPV-24 h), an effect that waned 24 h after the injection (+19%, *p* = 0.9398 versus vehicle-24 h).

A single injection of α-PVP, instead, did not alter *Npas4* mRNA levels at any time points analyzed (30 min: 0%, *p* > 0.999 versus vehicle-treated mice; 2 h: −4%, *p* = 0.9993 versus vehicle-treated mice; 24 h: +11%, *p* = 0.997 versus vehicle-treated mice). 

### 2.2. Molecular Analysis of the Effects of MDPV and α-PVP on Bdnf mRNA Levels 

#### 2.2.1. Hippocampus

Two-way ANOVA of *Bdnf* mRNA levels showed a main effect of treatment (F_2,43_ = 13.13, *p* < 0.0001), time after injection (F_2,43_ = 10.25, *p* = 0.0002) and a significant time × treatment interaction (F_4,43_ = 3.452, *p* = 0.0156; Figure 1 panel B). Post hoc analysis showed that *Bdnf* expression was differently modulated by the two cathinones depending on the time of sacrifice. In details, a single injection of MDPV increased *Bdnf* mRNA levels at 30 min (+26%, *p* = 0.0143 vs. vehicle-30 min; +30%, *p* = 0.001 vs. MDPV-24 h), 2 h (+22%, *p* = 0.0337 vs. vehicle-2 h; +28%, *p* = 0.0031 vs. MDPV-24 h), whereas it vanished 24 h later (−5%, *p* = 0.9981 vs. vehicle-24 h). When examining the effect of α-PVP, we found that it upregulated *Bdnf* mRNA levels at 30 min after treatment (+31%, *p* = 0.0017 vs. vehicle-30 min), while such increase disappeared at the other two time points investigated (2 h: +18%, *p* = 0.9992 vs. vehicle-treated mice; 24 h: +10%, *p* = 0.9046 vs. vehicle-treated mice).

#### 2.2.2. Frontal Lobe

Two-way ANOVA of *Bdnf* mRNA levels in the frontal lobe showed a main effect of treatment (F_2,43_ = 32.37, *p* < 0.0001), time after injection (F_2,43_ = 52.61, *p* < 0.0001) and a significant time × treatment interaction (F_4,43_ = 11.94, *p* < 0.0001; Figure 2 panel B). *Bdnf* expression appears to be modulated by the type of cathinone and the time of sacrifice after the single injection. A single injection of MDPV or α-PVP did not alter *Bdnf* mRNA levels 30 min or 2 h after drug exposure (MDPV: +26%, *p* = 0.5784 30 min vs. vehicle-30 min; +34%, *p* = 0.1811 2h vs. vehicle-2h; α-PVP: −1%, *p* = 0.9999 30 min vs. vehicle-30 min; −11%, *p* = 0.9988 2h vs. vehicle-2h), whereas both cathinones strongly upregulated the neurotrophin mRNA levels 24 h later (MDPV: +120%, *p* < 0.0001 versus vehicle-24 h; +96%, *p* < 0.0001 versus MDPV-30 min; +87%, *p* < 0.0001 versus MDPV-2h; α-PVP:+106%, *p* < 0.0001 versus vehicle-24 h; +109%, *p* < 0.0001 versus α-PVP-30 min; +118%, *p* < 0.0001 versus α-PVP-2h).

### 2.3. Molecular Analysis of the Effects of MDPV and α-PVP on vGAT1/vGluT1 Ratio 

#### 2.3.1. Hippocampus

**Figure 3:**
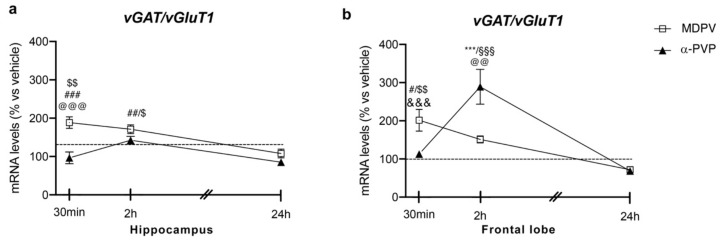
Time-dependent changes in *vGAT/vGluT1* mRNA levels in the hippocampus (**a**) and frontal lobe (**b**) of mice following a single IP injection of MDPV (1 mg/kg) or α-PVP (1 mg/kg). Mice were sacrificed at three different time points, i.e., 30 min, 2 h, and 24 h after the drug administration. Results are expressed as percentages relative to vehicle-treated group and presented as mean ± standard error of the mean (SEM). *** *p* < 0.001 α-PVP versus vehicle- treated mice; ^#^ *p* < 0.05, ^##^ *p* < 0.01, ^###^ *p* < 0.001 MDPV versus vehicle- treated mice; ^$^ *p* < 0.05, ^$$^ *p* < 0.01 MDPV versus MDPV-24 h; ^@@^ *p* < 0.01, ^@@@^ *p* < 0.001 MDPV versus α-PVP; ^&&&^ *p* < 0.001 α-PVP versus α-PVP-2h; ^§§§^ *p* < 0.001 α-PVP versus α-PVP-24 h (two- way ANOVA followed by Tukey’s multiple comparisons test).

Two-way ANOVA showed a main effect of treatment (F_2,43_ = 19.82, *p* < 0.0001), time after injection (F_2,43_ = 6.353, *p* = 0.0038) and a significant time × treatment interaction (F_4,43_ = 4.86, *p* = 0.0025) on *vGAT/vGluT1* ratio (Figure 3 panel A). In fact, a single injection of MDPV up-regulated *vGAT/vGluT1* ratio levels 30 min (+89%, *p* = 0.0003 vs. vehicle-30 min; +92%, *p* < 0.0001 vs. α-PVP-30 min; +70%, *p* = 0.0035 vs. MDPV-24 h), 2 h (+73%, *p* = 0.0023 vs. vehicle-2h; +58%, *p* = 0.0316 vs. MDPV-24 h), an effect that waned 24 h after the injection (+8%, *p* = 0.999 versus vehicle-24 h). Of note, a single injection of α-PVP did not alter *vGAT/vGluT1* ratio at the three time points investigated (30 min: −4%, *p* > 0.9999 versus vehicle-30 min; 2 h: +44%, *p* = 0.2075 versus vehicle-2h; 24 h: −16%, *p* = 0.9898 versus vehicle-24 h). 

#### 2.3.2. Frontal Lobe

Two-way ANOVA showed a main effect of treatment (F_2,42_ = 6.345, *p* = 0.0039), time after injection (F_2,42_ = 18.55, *p* < 0.0001) and a significant time × treatment interaction (F_4,43_ = 13.24, *p* < 0.0001; Figure 3 panel B). As observed for both *Npas4* and *Bdnf* mRNA levels, the profile of *vGAT/vGluT1* ratio strictly depended on both the type of drug and the time of sacrifice. Post hoc test revealed that a single injection of MDPV upregulated *vGAT/vGluT1* ratio 30 min (+101%, *p* = 0.0415 versus vehicle-30 min), an effect that waned 2 and 24 h after the injection (2 h: +52%, *p* = 0.6385 versus vehicle-2h; 24 h: −30%, *p* = 0.9816 versus vehicle-24 h). Conversely, a single injection of α-PVP did not alter *vGAT/vGluT1* ratio 30 min or 24 h after drug exposure (30 min: +13%, *p* > 0.9999 versus vehicle-30 min; 24 h: −33%, *p* = 0.9640 versus vehicle-24 h), whereas we observed a marked induction of such ratio 2 h after the injection (+192%, *p* < 0.0001 versus vehicle-2h).

## 3. Discussion

Our findings indicate that a single, low dose administration of the two synthetic cathinones MDPV and α-PVP is sufficient to alter the expression of molecular markers of neuroplasticity in the adult mouse brain. Of note, these drugs exhibit different effects on such markers, an effect that varies depending upon the brain region investigated and the time of sacrifice. These results indicate the substantial danger of the exposure to these synthetic cathinones that are normally taken at higher dosages and for prolonged periods in humans [39].

We found that MDPV, but not α-PVP, enhances *Npas4* mRNA levels in both the frontal lobe and hippocampus early after the injection of the drug (30 min); such effect persists for at least 2 h, and it vanishes 24 h later. Since NPAS4 is known to be critical for the development of GABAergic synapses, from these data we may infer that a single, low dose injection of MDPV, but not α-PVP, is sufficient to influence the homeostasis of GABAergic neurons. We next analyzed the ratio between the vesicular GABA transporter *vGAT* and the vesicular glutamate transporter *vGluT1,* in order to have a more comprehensive idea of the balance between inhibitory and excitatory neurotransmission. We found a similar trend in both the brain regions herein analyzed with some peculiarities that depend upon the time of sacrifice. In fact, in the hippocampus, in line with *Npas4* expression, MDPV, but not α-PVP, induced a rapid increase of such ratio (30 min) that persisted for 2 h whereas no effects of α-PVP were observed. In the frontal lobe, instead, MDPV raised such ratio rapidly (30 min), but then it declined back to control levels whereas α-PVP showed a peak of induction of *vGAT*/*vGluT1* ratio 2 h after the injection that vanished at the later time point. These data indicate that, early after the injection of these cathinones, MDPV, but not α-PVP, is able to rapidly activate the GABAergic over the glutamatergic system in the hippocampus, whereas α-PVP takes longer to sustain a similar response. Taken together, these results suggest that both cortical and hippocampal cells activate the GABAergic system early after the injection; however, the more rapid increase evoked by MDPV indicates that exposure to this compound is more demanding for the cell that immediately activates a defensive strategy by potentiating the inhibition of cell firing. It is interesting to note that, 24 h after the single injection, the balance between inhibitory and excitatory signaling is reestablished in both the brain regions investigated suggesting that both hippocampal and cortical cells mount an efficient response to MDPV or α-PVP to preserve cell homeostasis. These data suggest that the GABAergic system is indeed a target of the rapid action of cathinones and that a difference exists between these two brain regions in their ability to maintain a balance between cathinone-induced excitation and inhibition.

To deepen our knowledge on the effects of cathinones on cortical and hippocampal neuroplasticity, we investigated the neurotrophin Bdnf, whose expression has been previously shown to be regulated by the psychostimulant cocaine following single or repeated treatment [11,56,57,58,59,60,61,62]. Interestingly, under our experimental conditions, expression of the neurotrophin *Bdnf* follows a different pattern of activation when compared to *Npas4* depending on the time point analyzed. In the frontal lobe, MDPV, but not α-PVP, increases total *Bdnf* and *Npas4* mRNA levels at early time points whereas, 24 h after the single injection, both drugs show a concordant pattern of expression by markedly up-regulating *Bdnf* mRNA levels, independently from *Npas4* activation. A different picture is observed in the hippocampus where both drugs increase *Bdnf* mRNA levels 30 min and 2 h later, an effect that vanishes 24 h later. These findings indicate that MDPV and α-PVP display different effects on *Bdnf* gene expression that are brain-region-dependent. 

Since NPAS4 is a transcription factor that controls *Bdnf* mRNA level in an activity-dependent manner [3], we can infer that the increase in *Bdnf* mRNA levels observed in the frontal lobe 30 min and 2 h after MDPV exposure may be due, at least partially, to the relative increase of *Npas4* mRNA levels. Interestingly, such correlation is lost when animals are sacrificed 24 h after the single injection. The discrepancy between *Npas4* and *Bdnf* mRNA levels between early after the injection and 24 h suggests that other factors may have come into play to sustain *Bdnf* mRNA levels. For instance, the increase of *Bdnf* mRNA levels in both MDPV- and α−PVP-exposed mice may be indicative of a neurotrophic, neuroprotective response of the cell to cathinone exposure; in fact, we have previously shown that cocaine is able to promote *Bdnf* upregulation following a single exposure to the psychostimulant [53,56,63]. The different profile of *Bdnf* induction promoted by MPDV and α-PVP again sustains the possibility that the acute injection of MDPV generates a faster activation of the cell in comparison to α-PVP. Of note, *Npas4* has been reported to exhibit a neuroprotective effect, by promoting the survival of hippocampal neurons in response to excitatory stimulation [64], suggesting that both genes may cooperate to mount a rapid neuroprotective response to cathinones. Finally, these findings suggest that *Bdnf* mediates a portion of the inhibitory effects of *Npas4* on the GABAergic synapse, but that additional *Npas4* targets may also contribute. 

The brain is extremely vulnerable to substance-induced toxicity because of its high metabolic activity. Indeed, exposure to these stimulants may cause neurological adverse effects, increased body temperature and induce cognitive impairments. However, we must also recognize how it is undoubtedly difficult to ascribe a specific adverse effect to a specific stimulant as, in most cases, we are dealing with neurotoxicity due to polydrug intoxication [65]. We believe that the changes in neuroplasticity that we have pointed out, by showing an impairment of cortical and hippocampal homeostasis, might contribute to cathinone-induced neurotoxicity.

In conclusion, we hypothesize that *Npas4* up-regulation caused by the excitatory input promoted by cathinones may attempt to reduce cell activity, providing a negative feedback mechanism to preserve the homeostatic balance between excitation and inhibition. This is reinforced by the increased ratio *vGAT/vGluT1* and by the accompanying neuroprotective response caused by *Bdnf* up-regulation. These results clearly show that a single administration of a low dose of MDPV and α−PVP, pharmacologically active in mice [52] and corresponding to a light dose in humans, can dysregulate cortical and hippocampal homeostasis allowing to hypothesize that abuse of these cathinones at much higher doses could have an even more profound impact on neuroplasticity in humans.

## 4. Materials and Methods

### 4.1. Animals

Male ICR (CD-1^®^) mice weighing 25–30 g (Centralized Preclinical Research Laboratory, University of Ferrara, Italy) were group housed (5 mice per cage; floor area per animal was 80 cm^2^; minimum enclosure height was 12 cm), exposed to a 12:12-h light-dark cycle (light period from 6:30 am to 6:30 pm) at a temperature of 20–22 °C and humidity of 45–55% and were provided ad libitum access to food (Diet 4RF25 GLP; Mucedola, Settimo Milanese, Milan, Italy) and water. Experimental protocols performed in the present study were in accordance with the Guide for the Care and Use of Laboratory Animals as adopted and promulgated by the European Communities Council Directive of September 2010 (2010/63/EU) and were approved by Italian Ministry of Health (license n. 335/2016-PR) and by the Ethics Committee of the University of Ferrara. Moreover, adequate measures were taken to minimize the number of animals used and their pain and discomfort.

### 4.2. Drug Preparation and Dose Selection

MDPV and α-PVP were purchased from LGC Standards (LGC Standards S.r.l., Sesto San Giovanni, Milan, Italy). Drugs were initially dissolved in absolute ethanol (final concentration was 2%) and Tween 80 (2%) and brought to the final volume with vehicle (0.9% NaCl). The solution made of ethanol, Tween 80 and vehicle was also used as the vehicle. 

The 1 mg/kg dose of MDPV and α-PVP was chosen based on previous studies in mice [52]. Moreover, using specific interspecies dose scaling [66], 1 mg/kg dose is equivalent to a light dose in human (~4.86 mg and ~5.67 mg, in a human weighing 60–70 kg, respectively) as reported on internet experiences among users [39,67,68,69].

### 4.3. Analysis of Gene Expression 

Fifty-eight mice were treated with vehicle (n = 16), 1 mg/kg of MDPV (n = 18) or 1 mg/kg of α-PVP (n = 18) and sacrificed 30 min (30 min), 2 h (2 h) or 24 h (24 h) after the single injection. The dose of 1 mg/kg of MDPV and α-PVP was chosen based on a previously published behavioral effects and on changes in immediate early genes expression in a dose–response study [52]. At the scheduled times of sacrifice, mice were killed by cervical dislocation, their brains were quickly removed and the brain regions of interest were immediately dissected out, frozen on dry ice, and stored at −80 °C until being processed. Dissections were performed according to the mice atlas of Paxinos and Franklin [70]; frontal lobe (approximately from Bregma +2.58 to +1.70) was dissected from 2 mm coronal section while the hippocampi were dissected according to Spijker [71].

Total RNA was isolated by single step guanidinium isothiocyanate/phenol extraction using PureZol RNA isolation reagent (Bio-Rad Laboratories, Segrate, Milan, Italy) according to the manufacturer’s instructions and quantified by spectrophotometric analysis. Following total RNA extraction, the samples were processed for real-time reverse transcription polymerase chain reaction (real time RT-PCR) to assess mRNA levels. Briefly, an aliquot of each sample was treated with DNase to avoid DNA contamination. RNA was analyzed by TaqMan qRT-PCR instrument (CFX384 real time system, Bio-Rad Laboratories) using the iScriptTM one-step RT-PCR kit for probes (Bio-Rad Laboratories). Samples were run in 384 wells formats in triplicate as multiplexed reactions. Data were analyzed with the comparative threshold cycle (∆∆Ct) method using 36B4 as reference gene [72]. The primer efficiencies were experimentally set up for each couple of primers. Thermal cycling was initiated with an incubation at 50 °C for 10 min (RNA retrotranscription) and then at 95 °C for 5 min (retrotranscriptase inactivation). After this initial step, 39 cycles of PCR were performed. Each PCR cycle consisted of heating the samples at 95 °C for 10 s to enable the melting process and then for 30 s at 60 °C for the annealing and extension reaction.

Primers and probe for *total Bdnf, Npas4, vGluT1, vGAT* and *36B4* were purchased from Eurofins MWG-Operon. Their sequences are shown below: 

*total Bdnf*: forward primer 5′-AAGTCTGCATTACATTCCTCGA-3′, reverse primer 5′-GTTTTCTGAAAGAGGGACAGTTTAT-3′, probe 5′- TGTGGTTTGTTGCCGTTGCCAAG-3′;

*Npas4*: forward primer 5′- TCATTGACCCTGCTGACCAT -3′, reverse primer 5′- AAGCACCAGTTTGTTGCCTG -3′, probe 5′- TGATCGCCTTTTCCGTTGTC-3′;

*vGluT1*: Forward primer 5′-ACTGCCTCACCTTGTCATG-3′, Reverse Primer 5′-GTAGCTTCCATCCCGAA ACC-3′, Probe 5′-CTTTCGCACATTGGTCGTGGACAT T-3′; 

*vGAT*: Forward primer 5′-ACGACAAACCCAAGAT CACG-3′, Reverse Primer 5′-GTAGACCCAGCACGAA CATG-3′, Probe 5′-TTCCAGCCCGCTTCCCACG-3′; 

*36B4*: forward primer 5′-TTCCCACTGGCTGAAAAGGT-3′, reverse primer 5′-CGCAGCCGCAAATGC-3′, probe 5′-AAGGCCTTCCTGGCC GATCCATC-3′.

### 4.4. Data and Statistical Analysis

Molecular data were collected in individual animals (independent determinations) and are presented as means ± standard errors. Changes produced by treatment and time after the injection alone as well as by their combination were analyzed using a two-way analysis of variance (ANOVA), with treatment and time after injection as independent variables. When appropriate, further differences between groups were analyzed by Tukey’s multiple comparisons test. Statistical significance was assumed at *p* < 0.05. No changes were observed in the expression levels of the targets analyzed in both brain areas among vehicle-treated mice sacrificed at 30 min, 2 h and 24 h.

The statistical analysis was performed with the program Prism software (GraphPad Prism, San Diego, CA, USA).

## Data Availability

The data presented in this study are available on request from the first (Lucia Caffino) and corresponding author (Fabio Fumagalli) for researchers of academic institutes who meet the criteria for access to the confidential data.
